# QTL mapping and identification of candidate genes using a genome-wide association study for heat tolerance at anthesis in rice (*Oryza sativa* L.)

**DOI:** 10.3389/fgene.2022.983525

**Published:** 2022-09-15

**Authors:** Changmin Hu, Jianhua Jiang, Yulong Li, Shaojie Song, Yu Zou, Chunyu Jing, Ying Zhang, Dezheng Wang, Qiang He, Xiaojing Dang

**Affiliations:** ^1^ Institute of Rice Research, Anhui Academy of Agricultural Sciences, Hefei, China; ^2^ College of Agronomy, Anhui Agricultural University, Hefei, China; ^3^ Institute of Crop Research, Anhui Academy of Agricultural Sciences, Hefei, China; ^4^ State Key Laboratory of Hybrid Rice, Hunan Hybrid Rice Research Center, Changsha, China

**Keywords:** anthesis, heat tolerance, genome-wide association study, qRSF9.2, high and stable yield

## Abstract

Heat tolerance (HT) of rice at anthesis is a key trait that ensures high and stable yields under heat stress. Finding the quantitative trait loci (QTLs) and gene loci controlling HT is crucial. We used relative spikelet fertility (RSF) as a measure of HT. The phenotypic values of RSF in 173 rice accessions were investigated in two environments and showed abundant variations. We performed a genome-wide association study on RSF using 1.2 million single nucleotide polymorphisms (SNPs). Five QTLs were significantly associated with RSF were identified, four were found in previously reported QTLs/genes, and one was novel. The novel QTL *qRSF9.2* was mapped into the 22,059,984-22,259,984 bp region, which had 38 positional candidate genes. By combining the linkage disequilibrium analysis, the QTL region was narrowed to 22,110,508–22,187,677 bp, which contained 16 candidate genes. Among them, only gene *LOC_Os09g38500* contained nonsynonymous SNPs that were significantly associated with RSF. In addition, accessions with large and small RSF values had corresponding respective high and low gene expression levels. Furthermore, the RSF of the CC allele was significantly higher than that of the TT allele. Hap 2 and Hap 3 can increase heat tolerance by 7.9 and 11.3%, respectively. Our results provide useful information that recommends further cloning of *qRSF9.2* and breeding heat-tolerant rice varieties by marker-assisted selection.

## Introduction

Globally, rice is a vital food crop and is the staple food of the majority of the world’s population (Pan et al., 2020). Heat damage caused by climate warming is one of the main abiotic stresses affecting rice production. In the reproductive growth period, especially in the flowering period, rice plants are sensitive to high temperature, which hinders their flowering and fertilization and thereby reduces the seed setting rate and yield (Nubankoh et al., 2020).

Recently, heat damage frequently occurred in the rice-planting area. For example, in 2006 and 2007, the rice-planting areas of the Yangtze River Valley in China experienced unusually high temperatures for >20 d, which lead to a 50% reduction in the rice seed setting rate in some areas (Xiao et al., 2011). In 2013 and 2017, during the rice booting and flowering periods, the high temperature weather in the Yangtze River Valley in China continued for >20 d. In 2017, a 2.67×10^5^ hm^2^ of rice was damaged by heat in the Anhui Region (Zhang et al., 2020). Studies have shown that for every 1°C the air temperature increases, the rice yield will decrease by 3.2% (Zhao et al., 2017). Gourdji et al. (2013) predicted that by 2030, about 16% of global rice production will experience heat stress above the critical point for at least 5 d during reproductive growth. However, the main rice varieties that are currently used are generally sensitive to high temperature, and so improving their heat tolerance has become an urgent task (Shi et al., 2018). Bhardwaj et al. (2022) indicated that it is necessary to use an ‘omics’ approaches in developing combined drought and heat tolerance in food crops.

Heat tolerance is an extremely complex quantitative trait in rice, which is controlled by many genes (Ishimaru et al., 2010). To date, 205 quantitative trait loci (QTLs) controlling heat tolerance-related traits have been identified, which were located on all 12 chromosomes. These QTLs were detected based on different populations, such as F_2_ (Asako et al., 2007; Ye et al., 2012, 2015a,b; Zheng et al., 2017; Nubankoh et al., 2020), F_2:3_ (Chen et al., 2021), doubled haploid (DH) (Cao et al., 2002), backcross inbred lines (BILs) (Zhu et al., 2006; Tazib et al., 2015; Cao et al., 2020), recombinant inbred lines (RILs) (Tabata et al., 2007; Chen et al., 2008; Jagadish et al., 2010; Xiao et al., 2011; Wada et al., 2015; Shanmugavadivel et al., 2017; Kilasi et al., 2018; Li et al., 2018), chromosome segment substitution lines (CSSLs) (Zhao et al., 2016; Zhu et al., 2017) and natural population (Pan et al., 2021), a different growth and development period from seedling stage to grain filling stage, different ways of heat stress, such as natural or artificial high temperature stress, different evaluation indexes, such as heading and flowering time, and indexes related to yield/seed setting rate and quality. Among them, 40 QTLs were detected at the heading and anthesis stages, which were most susceptible to high temperature stress (Jagadish et al., 2010; Cheng et al., 2012; Ye et al., 2012; Ye et al., 2015a; Ye et al., 2015b; Shanmugavadivel et al., 2017; Li et al., 2018; Nubankoh et al., 2020; Chen et al., 2021).

During this period, several QTLs or genes for heat tolerance were successively cloned at the seedling stage. *OsTT1* (Thermo-tolerance 1) is the first cloned QTL for heat tolerance of rice at the seedling stage. The *OsTT1* protein protects cells from heat stress by allowing more efficient elimination of cytotoxic denatured proteins and more effective maintenance of heat-response processes to reduce the yield loss caused by heat stress (Li et al., 2015). *TOGR1* (Thermotolerant growth required 1) encodes a DEAD-box RNA helicase regulated by both temperature and the circadian clock and is associated with the pre-rRNA processome at high temperatures, which regulates the thermotolerant growth of rice (Wang et al., 2016). *OsHTAS* (Heat tolerance at seedling stage) encodes a ring finger ubiquitin ligase and modulated hydrogen peroxide-induced stomatal closure to enhance heat tolerance at the seedling stage in rice (Liu et al., 2016). Chen et al. (2019) identified *AET1*, a tRNA^His^ guanylytransferase, which regulates auxin signaling in response to high temperatures and the environmental temperature response in rice. Cao et al. (2022) identified *HTH5* from *Oryza rufipogen* Griff, which reduced reactive oxygen species accumulation at high temperatures by increasing the level of the heat-induced pyridoxal 5′-phosphate, increasing pollen vitality, and thereby improving spikelet fertility. The heat tolerance QTL *qHTSF4.1* was introgressed into the IR64 background using the molecular marker-assisted selection method and the near isogenic lines IR64HT_4_ were developed. The seed setting rate of IR64HT_4_ was significantly higher than that of its recurrent parent IR64 under high temperature (Ye et al., 2022).

Therefore, screening and identifying germplasm resources, mining more genes, and breeding new rice varieties with greater heat tolerance are the main ways of reducing heat stress and stabilizing grain yield. Here, we investigated the phenotype values of the relative seed setting rate in 173 rice materials and performed a genome-wide association study (GWAS) to mine markers associated with heat tolerance and predict candidate genes. These results provide a theoretical foundation for elucidating the genetic mechanism of heat tolerance at the heading and flowering stage and a material foundation for breeding new heat-tolerant rice varieties.

## Materials and methods

### Plant materials and field planting

A set of 173 rice materials were obtained from Dang et al. (2020), of which, 134 were from China, including 74 improved and 60 local varieties, and 39 from Southeast Asian countries. The details of these rice samples, including their names, origins, and sequence IDs, are summarized in Supplementary Table S1. Fengliangyou 4hao was selected as the high temperature tolerant control based on its high heat tolerance, high yield, and wide use, and Yanhui 559 was selected as the high temperature sensitive control. These materials were grown at the Experimental Station of Anhui Academy of Agricultural Sciences (Hefei, Anhui Province, China) in 2020 and 2021, respectively. Routine field management guidelines followed. The seeds were planted on May 10, 2020. On June 8, the seedlings were transplanted into a 20 cm × 30 cm plastic basin loaded with substrate (1 kg) and natural air-dried soil (3 kg). One plastic basin was considered a single plot. Each plant sample was planted in four plots with four plants. A completely random design was used regardless of natural or heat treatment. The same process was performed in 2021.

### High temperature stress treatment

The high temperature stress treatment was conducted like Zhang et al. (2020). For one sample, > 10 panicles heading on the same day were selected and labeled with a plastic tag. At 09:30 in the morning, two plots with the plastic tag were put into the intelligent greenhouse of Rice Research Institute of Anhui Academy of Agricultural Sciences. The temperature settings for each time period are as follows: 07:01-09:30 for 32.5 °C; 09:31-14:30, 40.0 °C; 14:31-17:00, 36.5 °C; 17:01-21:00, 32.0 °C; and 21:01-07:00, 29.0 °C. The relative humidity in the greenhouse was controlled at 60–70%. The treatment continued for 5 d. The treated plants were removed from the greenhouse at 09:30 on the sixth day and placed under natural conditions to grow until mature. In addition, the other two plots with labeled tags were used as controls to grow to maturity under natural conditions.

### Trait investigation and heat resistance evaluation

On the 20th day after the high temperature treatment, 10 panicles with labeled tags were taken from the control and treatment groups. The total number of spikelets (TNS) and the number of filled spikelets (NFS) of each panicle were counted. The spikelet fertility (SF) for a single panicle was calculated. The formula was SF (%) = (NFS/TNS) × 100. The SF under natural conditions was recorded as SF_n_. The SF in the high temperature condition was recorded as SF_ht_. The relative spikelet fertility (RSF) was calculated as follows: RSF (%) = (SF_ht_/SF_n_) × 100. The RSF was used as the measure for heat tolerance at anthesis in rice.

### Genotypic data

The sequences of the accessions are available at the NCBI Sequence Read Archive with accession number PRJNA554986 (Dang et al., 2020). All paired-end sequence reads were aligned against the Os-Nipponbare-Reference-IRGSP 1.0 with Bowtie 2 (Li and Durbin, 2010). More than 95% of the reads with a mapping score >60 were found and mapped to the reference genome. The SNP calling was performed by HaplotypeCaller from GATK 3.8-0. The missing genotype data was completed with Beagle v4.1 (Browning and Browning, 2009). A total of 1,224,254 SNPs with a minor allele frequency (MAF) > 5% and a missing rate <20% were obtained to estimate population structure, kinship coefficients, and perform GWAS.

### Population structure analysis

STRUCTURE 2.3.4 software (Pritchard et al., 2000) was used to analyze the population structure with a burn-in of 50,000 replicates and 100,000 Markov chain Monte Carlo iterations. Ten repeats for each K value (*K* = 1-10) were performed. The EVANNO method with STRUCTURE HARVESTER (Earl and vonHoldt, 2012) was used to analyze the results, and CLUMPP V 1.1.2 (Jakobsson and Rosenberg, 2007) was used to permute run clusters. The ΔK was calculated to determine the suitable K value. We used DISTRUCT (Rosenberg, 2004) to plot results. The non-admixed individual in each subgroup with Q-matrix assignment was >0.85. PHYLIP 3.2 (Felsenstein, 1993) was used to construct the neighbor-joining tree and MEGA 5.0 (Tamura et al., 2011) was used to display the tree. The smartpca program from gcta64 (Price et al., 2006) was used to perform principal component analysis and plot the first two principal components in two dimensions.

### Genome-wide association analysis

The kinship matrix was calculated using TASSEL 5.2.1 (Bradbury et al., 2007). GWAS was performed using the R package Genomic Association and Prediction Integrated Tool (Lipka et al., 2012) based on a mixed linear model. Referring to the methods reported by Yang et al. (2014), the region was recognized as a QTL when the associations loci exceeded the *p*-value thresholds (<1 × 10^-4^) with clear peak-like signals (≥3 significant SNPs) and they were in the 200 kb region of the leading SNP. The linkage disequilibrium (LD) heatmaps surrounding peaks in the GWAS (Shin et al., 2006) were constructed using the R package “LDheatmap.” We used the R package qqman (Turner, 2014) to draw the manhattan and quantile-quantile plots. The Benjamini and Hochberg (1995) correction method was used to determine the genome-wide significance thresholds of the GWAS, which was 1.0 × 10^-4^ at a nominal level of 0.05.

### Candidate gene analysis

The candidate genes were predicted within a 200 kb genomic region, i.e., ± 100 kb of the significant leading SNP of the QTL based on the Rice Genome Annotation Project MSU7 database (http://rice.plantbiology.Msu.edu). LD analysis was performed with Haploview to define LD blocks surrounding significant SNPs (Barrett et al., 2005). The causal gene for each locus was decided based on the gene annotation and expression analysis.

### Quantitative reverse transcription PCR analysis of candidate genes

Three accessions with high RSF values were subject to 38 °C heat stress from 09:30 a.m. to 15:30 p.m. during the day for 3 d in a phytotron, and three plants were used for each sample. The same was done for three accessions with low RSF. After a 3 d heat treatment, we sampled the panicle tissues at different times, i.e., at 08:30 a.m., 09:30 a.m., 10:30 a.m., 11:30 a.m., 12:30 p.m., 13:30 p.m., 14:30 p.m., 15:30 p.m., 16:30 p.m., and 17: 30 p.m., and we named the sampling periods in order from No. 1 to 10.

The total RNA was extracted using the TianGen Pure Plant Plus Kit (TianGen Biotech Co., Ltd., Beijing) following the manufacturer’s protocol. We used the HisScript II Reverse Transcriptase system (Vazyme Biotech Co., Ltd., Nanjing, China) to synthesize the first-strand cDNA with random oligonucleotides. The UBQ rRNA gene was used as an internal control. The 96-well thermocycler (Roche Applied Science LightCycler 480, https://lifescience.roche.com/) was used to perform the real-time quantitative PCR (qRT-PCR) by SYBR Green (Vazyme). The cycling conditions were as follows: first, denaturation occurred at 95 °C for 5 min; second, an amplification and quantification program with 40 cycles (a single fluorescence measurement) at 95 °C for 10 s, 60 °C for 30 s, and 72 °C for 60 s occurred; third, the melting curve (continuous fluorescence measurement) occurred at 60-95 °C with a heating rate of 0.1 °C sec^−1^; and finally, a cooling step occurred at 40 °C. Three biological replicate experiments were conducted. The primers used for qRT-PCR are listed in Supplementary Table S2. The transcript levels of gene expression were calculated (Livak and Schmittgen, 2001) following the equation exp = 2^−ΔCt^, where ΔCt = Ct_target gene_ − Ct_UBQ rRNA_.

### Haplotype analysis

Using the RiceVarMap (http://ricevarmap.ncpgr.cn/) and China Rice Data Center (https://www.ricedata.cn/) databases, the haplotypes of candidate genes were determined. When haplotypes were represented by more than 20 accessions, we considered it a haplotype.

### Data analysis

The statistical data analysis was conducted by the Statistix 8.0 software (Analytical Software, Tallahassee, Florida, USA). The random model was used for analysis of variance (ANOVA). The broad-sense heritability was calculated using the following formula based on the results described in the ANOVA table. The formula is H2_B_ (%) = [(MS_g_-MS_e_)/r]/[(MS_g_-MS_e_)/r + MS_e_]*100%. MS_g_ is the mean square of the genotype, MS_e_ is mean square of the error, and r is number of replicates.

## Results

### Phenotypic analysis of SF under natural and high temperature stress conditions

**FIGURE 1:**
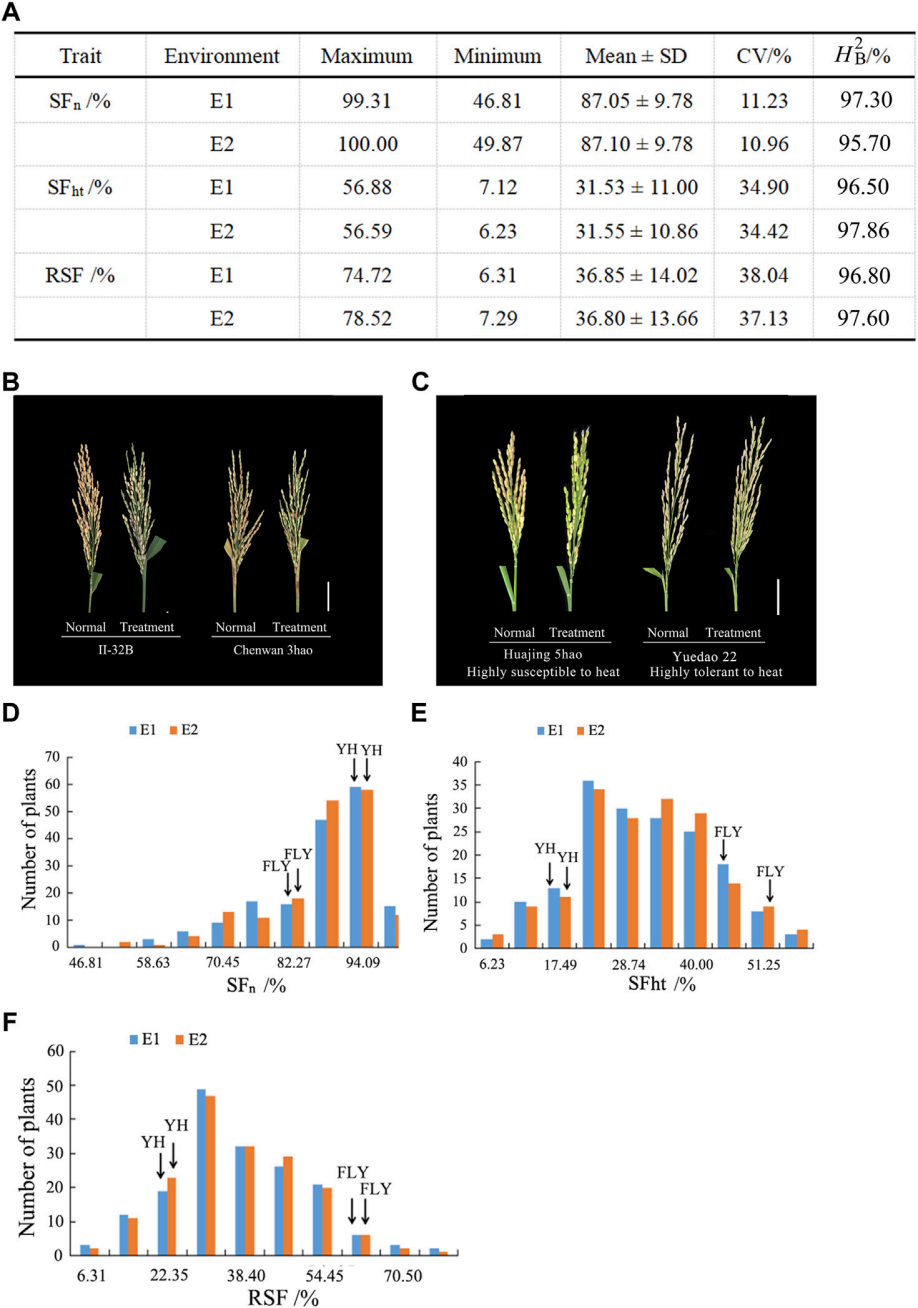
Phenotypic diversity of heat tolerance relative traits in rice. **(A)** Phenotypic statistics of heat tolerance relative traits in E1 and E2. **(B)** Panicle morphology of materials II-32B and Chenwan 3hao in the normal and treatment conditions, respectively. Scale bar, 5 cm. **(C)** Panicle morphology of material Huajing 5hao that is highly susceptible to heat and highly tolerant to heat material Shengtangqing in normal and treatment conditions, respectively. Scale bar, 5 cm. **(D)** Variation of SF_n_ among the 173 rice accessions in E1 and E2. **(E)** Variation of SF_ht_ among the 173 rice accessions in E1 and E2. **(F)** Variation of RSF among the 173 rice accessions in E1 and E2. H2_B_, the broad-sense heritability; SF_n_, spikelet fertility in normal conditions; SF_ht_, spikelet fertility in high temperature conditions; RSF, relative spikelet fertility.

The maximum, minimum, and mean values as well as coefficients of variation (CV) of heat tolerance-related traits are listed in Figure 1A. Figure 1B shows the SF of rice panicles for materials under natural and high temperature treatment conditions. The SF of II-32B was 83.36% under natural conditions and 37.49% under high temperature treatment with a difference value of 45.87%. The SF of Chenwan 3hao was 56.77% under natural conditions and 29.55% under the high temperature treatment with a difference value of 27.22%. These results suggest that RSF is a reliable measurement indicator of heat tolerance at the flowering stage in rice.

Figure 1C displays the rice panicle morphology of the genotypes that are highly tolerant and susceptible to heat. The maximum value of the SF_n_ was 100% and the minimum value was 46.81% among the two environments (Figures 1A,D). The CV ranged from 11.23 to 10.96% across the two environments. Figure 1D shows that the SF_n_ of Yanhui 559 was 92.44% in E1 and 93.42% in E2, and the SF_n_ of Fengliangyou 4hao was 80.64% in E1 and 82.15% in E2.

The maximum value of SF_ht_ was 56.88% and the minimum value was 6.23% among the two environments, and the CV ranged from 34.90 to 34.42% (Figures 1A,E). The SF_ht_ of Yanhui 559 was 18.59% in E1 and 20.03% in E2, and the SF_ht_ of Fengliangyou 4hao was 47.70% in E1 and 51.31% in E2 (Figure 1E).

For the RSF trait, the mean value was 36.85 ± 14.02% in E1 and 36.80 ± 13.66% in E2 with the CV ranging from 37.13 to 38.22% (Figures 1A,F). The RSF of Yanhui 559 was 20.10% in E1 and 21.44% in E2, with the RSF of Fengliangyou 4hao was 58.87% in E1 and 62.77% in E2 (Figure 1F). The average broad-sense heritability across two years was 96.5% for SF_n_, 97.18% for SF_ht_ and 97.20% for RSF (Figure 1A). The scatterplots of SF_n_, SF_ht_ and RSF across two years were conducted and shown in Supplementary Figure S1. Based on the results of analysis of variance for SF_n_, SF_ht_, and RSF, we found that there were significant differences among genotypes and no significant differences among the environments (Table 1). These results suggest that the traits of SF_n_, SF_ht_, and RSF were genetically stable and controlled by genotypes in two environments.

**TABLE 1:** The results of analysis of variance for the heat tolerance-related traits.

Traits	Source of variation	df	SS	MS	*F*-value	*F*_0.05_	*F*_0.01_
SF_n_/%	Genotypes	172	31782.0845	184.7796	56.0627**	1.31	1.47
	Environments	1	0.1920	0.1920	0.0583	3.89	6.76
SF_ht_/%	Genotypes	172	37022.0601	215.2445	69.8428**	1.31	1.47
	Environments	1	0.0270	0.0270	0.0088	3.89	6.76
RSF/%	Genotypes	172	60512.1241	351.8147	70.3505**	1.31	1.47
	Environments	1	0.0215	0.0215	0.0043	3.89	6.76

df, degrees of freedom; SS, sum of squares; MS, mean square.** Significant differences at *p* < 0.01. SF_n_, spikelet fertility under natural condition; SF_ht_, spikelet fertility under high temperature condition; RSF, relative spikelet fertility.

### Population structure

**FIGURE 2:**
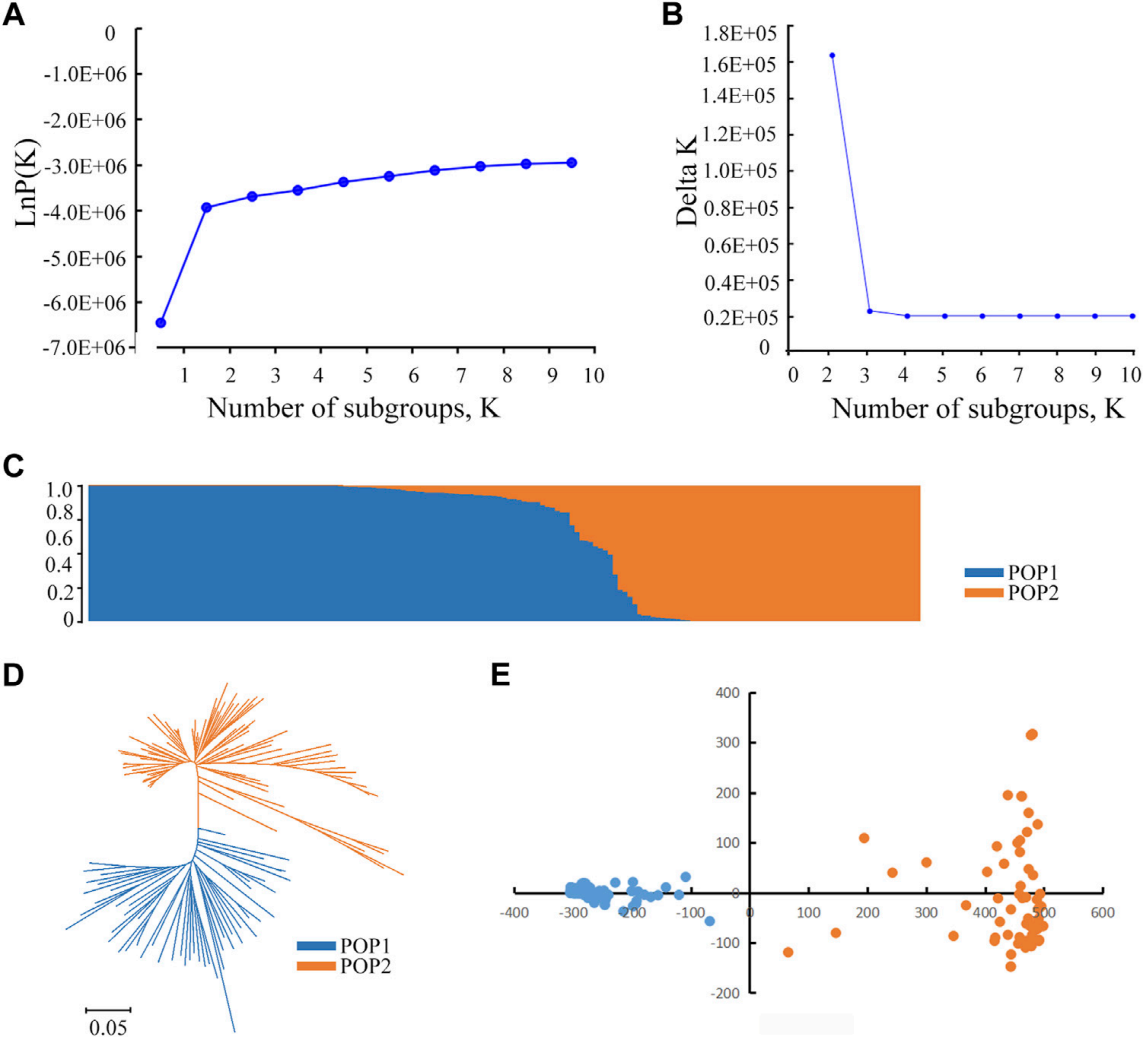
Population structure analysis of 173 rice accessions. **(A)** Changes of the mean LnP(K). A graph of *Y* axis means of mean LnP(K) and *X* axis means for the number of subgroups. **(B)** Changes in the mean delta K. A graph of *Y* axis means of mean delta K and *X* axis means for the number of subgroups. **(C)** The population structure based on a Bayesian model. Each vertical bar represents one accession, and the length of each vertical bar represents the ancestral proportion. **(D)** Phylogenetic tree of 173 accessions based on whole-genome SNPs. The scale bar at the bottom represents genetic distance. Accessions within different subgroups are displayed by different colors. **(E)** Principal component analysis showing the population structure in the diversity panel. The red lines indicate *Indica* accessions. The blue lines indicate *Japonica* accessions.

STRUCTURE was used to calculate the genetic composition of each variety. When *K* = 2, the inflection point appears on the broken line (Figure 2A). Then, the logarithmic likelihood value decreases as the K value increases. To determine subgroups, we calculated the ΔK value. When *K* = 2, ΔK reaches its maximum value (Figure 2B). Therefore, we divided the 173 rice varieties into two subgroups named POP1 and POP2 (Figure 2C). The materials were divided into a special subgroup with a Q value > 0.90. The results showed that POP1 contained 91 materials that mainly belonged to *Japonica*, and POP2 contained 59 materials that mainly belong to *Indica*. The remaining 23 accessions could not be divided into fixed subgroups and were considered a mixed group. Based on Nei’s genetic distance (Nei et al., 1983), the neighbor trees showed that 173 materials were divisible into two subgroups (Figure 2D). According to the eigenvector distribution, the 173 accessions could be divided into two clusters by principal component analysis (Figure 2E). The first and second principal component accounted for 7.40 and 2.48% of the total genetic variation, respectively. These results indicate that the population can be reliably divided into two subgroups.

### Identification of QTLs for heat tolerance-related traits by GWAS

**FIGURE 3:**
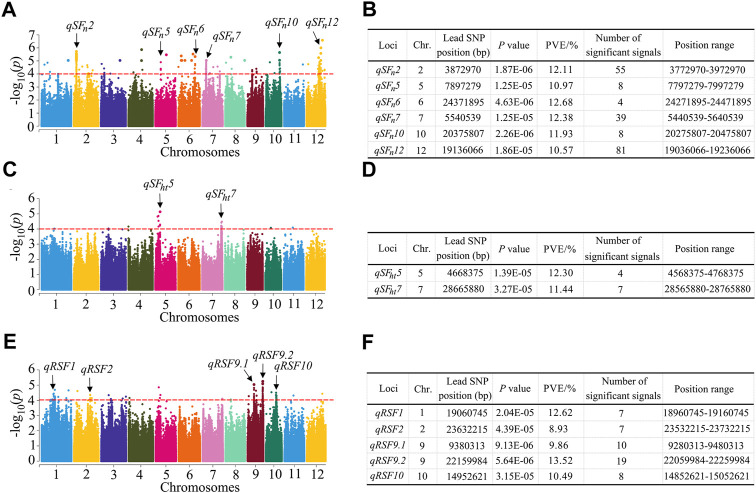
Identification of QTLs by GWAS in rice. **(A)** Manhattan plots for SF_n_ in the whole population of rice accessions. **(B)** Information of the identified QTL for SF_n_. **(C)** Manhattan plots for SF_ht_ in the whole population of rice accessions. **(D)** Information of the identified QTL for SF_ht_. **(E)** Manhattan plots for RSF in the whole population of rice accessions. **(F)** Information of the identified QTL for RSF. *Y* axis means negative log_10_ transformed *p*-values and dots above the red line show the significant SNPs in the QTL region. The black arrows indicate the QTL identified. SF_n_, spikelet fertility in normal condition; SF_ht_, spikelet fertility in high temperature condition; RSF, relative spikelet fertility. PVE: phenotypic variation explained.

Based on the 1,224,254 SNP, we performed a GWAS to investigate the possible natural variation in heat tolerance of rice and constructed the Manhattan plots to illustrate the significant SNPs related to rice heat tolerance. When >3 SNPs exceeded the significance threshold value of 1 × 10^-4^, we considered the 200 kb interval of the leading SNP as a QTL. For the SF_n_ trait, a total of six QTLs were detected, which were located on chromosomes 2, 5, 6, 7, 10 and 12 (Figures 3A,B). There were 39 significant SNPs in the *qSF*_*n*_ *7* region. The phenotypic variation explained (PVE) ranged from 10.57 to 12.68%. For the SF_ht_ trait, we identified two QTLs located on chromosomes 5 and 7 (Figures 3C,D) with PVEs of 12.30 and 11.44%, respectively. For RSF, a total of five QTLs were identified, which were located on chromosomes 1, 2, 9 and 10 (Figures 3E,F). The PVE ranged from 8.93 to 13.52%. Among them, the largest number of significant SNPs (19) existed in the *qRSF9.2* region on chromosome 9, which was considered a major QTL for relative spikelet fertility. Next, we focused our investigation on *qRSF9.2*.

### Identification of candidate genes

**FIGURE 4:**
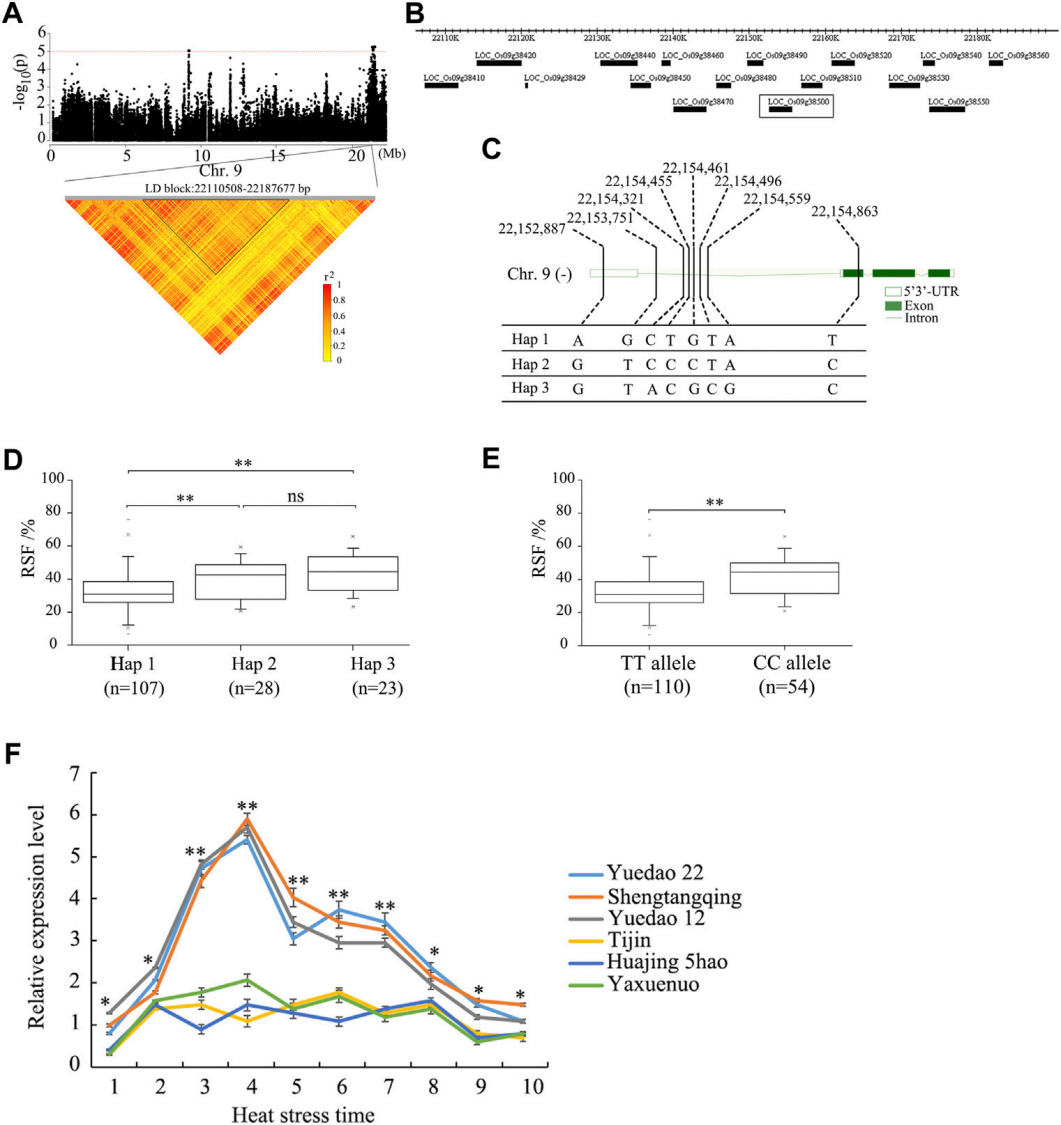
Identification of the candidate genes for the QTL *qRSF9.2* for relative spikelet fertility as determined by integrated analyses of GWAS and gene expression data in rice. **(A)** Identification of the haplotype block of *qRSF9*.2. *Y* axis means negative log_10_ transformed *p*-values and dots above the red line show the significant SNPs in the QTL region. Pairwise LD was determined by calculation of *r* ^*2*^ (the square of the correlation coefficient between SNP states). **(B)** Identification of candidate genes in the region of *qRSF9.2*. **(C)** Haplotypes of *LOC_Os09g38500* associated with relative spikelet fertility in rice. **(D, E)** Box-plots of relative spikelet fertility in accessions containing the different haplotypes **(D)** and elite alleles **(E)**. **(F)** Expression analysis of candidate gene *LOC_Os09g38500* in different materials under heat stress for various time periods. The relative expression values were normalized to the rice UBQ gene. The error bars indicate standard deviation, and asterisks indicate significant differences using the Student’s *t*-test (*p < 0.05, **p < 0.01). Numbers 1-10 on *X* axis indicate the sampling time at 08:30 a.m., 09:30 a.m., 10:30 a.m., 11:30 a.m., 12:30 p.m., 13:30 p.m., 14:30 p.m., 15:30 p.m., 16:30 p.m., and 17:30 p.m.

To further analyze the candidate genes in the chromosome region containing *qRSF9.2*, all potential candidates within 200 kb were analyzed (Figure 4A). A total of 38 positional candidate genes were identified for *qRSF9.2*. Combining the LD analysis, we determined the LD block region as 22, 110, 508–22, 187, and 677 bp, which contained 16 candidate genes (Figure 4B). Based on SNP information, 6 of the 16 genes contained nonsynonymous SNPs (Supplementary Tables S3, S4). However, only one nonsynonymous SNP was significantly associated with RSF by GWAS (Supplementary Table S3), which was located within the gene locus *LOC_Os09g38500.* The full length of *LOC_Os09g38500* is 2,936 bp, which includes three exons and three introns (Figure 4C). Gene *LOC_Os09g38500* encodes a 224 amino acid protein. The SNP polymorphisms occurred in the 3′-primer UTR, the intron, and the coding sequence of the gene. This resulted in the identification of three haplotypes (Figure 4C). The haplotype Hap 1 was associated with a smaller RSF, while haplotypes Hap 2 and Hap 3 were associated with a larger RSF (Figure 4D). The SNP site (22,154,863) causes a change from base C to base T at nt 170 in the cDNA sequence, which results in an amino acid change from arginine (A) to lysine (L) at amino acid 57. The average RSF values of 110 accessions carrying the TT allele was 33.2 ± 12.7%, while those with the CC allele had an RSF of 42.3 ± 11.1%. There were highly significant differences between the TT allele and CC allele at *p* < 0.01 (Figure 4E).

The relative expression levels of 16 candidate genes in the panicle between three accessions each with high or low RSF were analyzed under different durations of heat stresses through qRT-PCR. The qRT-PCR results showed no significant expression difference among 16 candidate genes except gene *LOC_Os09g38500* (Figure 4F, Supplementary Figure S2)*.* The expression of the CC allele in each of the three accessions (Yuedao 22, Shengtangqing, and Yuedao12) with large RSFs was significantly higher than that of the TT allele in each of the three accessions (Tijin, Huajing 5hao, and Yaxuenuo) with small RSFs (Figure 4F). The expression of *LOC_Os09g38500* in high-RSF accessions was significantly higher (*p* < 0.01) than that in low-RSF accessions at heat stress times ranging from 10:30 a.m. to 14:30 p.m. The expression of *LOC_Os09g38500* in the accessions with high RSF was higher (*p* < 0.05) than that in accessions with low RSF at a heat stress time ranging from 08:30 a.m. to 09:30 a.m. and from 15:30 p.m. to 17:30 p.m. (Figure 4F). The expression of *LOC_Os09g38500* in the accessions with large RSF showed gradual increases at heat stress timed from 08:30 a.m. to 11:30 a.m., reaching a maximum at 11:30 a.m. After 12:30 p.m., the expression of *LOC_Os09g38500* started to gradually decline (Figure 4F)*.* These results suggest that enhanced expression of the CC allele may increase RSF.

## Discussion

Presently, natural high temperatures in the field and artificial climate simulation methods are generally used to identify the heat tolerance of rice varieties. However, artificial climate chambers are more advantageous to the selection of heat-resistant rice varieties because of their environmental controllability. Kumar et al. (2016) evaluated the heat tolerance of the same rice materials in the field and under climate chamber conditions and found that the SF at high temperature field conditions was higher than that at high temperature conditions in a climate chamber. This difference may be caused by factors such as humidity, wind speed, and light intensity (Pan et al., 2015).

In this study, to improve the accuracy of identifying heat tolerance, we sowed 173 accessions in stages and marked the panicles under the heat temperature at the flowering stage. The heat tolerance of 173 rice cultivars were evaluated based on the SF of marked panicles. Although field identification is affected by climate change, it is more suitable for rice production and breeding practice and can accurately reflect the heat tolerance of rice (Zhao et al., 2016). In this study, the rice canopy temperature in the field was approximately 40 °C. The SF of different materials at high temperature was obviously different, but the average SF was 64%, which is much higher than that observed at 38 °C in a greenhouse by Lafarge et al. (2017). We found three materials (Yuedao 12, Yuedao 22, and Shengtangqing) with the same heat tolerance as Fengliangyou 4hao. Among them, Yuedao 12 and Yuedao 22 belong to breeding varieties. These varieties were suitable for planting in high temperature-prone areas to reduce the yield loss. Shengtangqing belongs to Taihu Valley local varieties. After a long-term adaptive evolution, these local varieties have rich genetic diversity and strong stress tolerance. Therefore, these materials can be used as donor parents for the future genetic improvement of heat tolerance.

We detected six QTLs for SF_n_, two for SF_ht_ and five for RSF (Figure 3). We then compared them with the previously reported QTLs through the Gramene website (http://www.gramene.org/markers/), BLAST (http://blast.ncbi.nlm.nih.gov/Blast.cgi), and the China Rice Data Center database (http://www.ricedata.cn/gene/list/1499.htm). The position range of one QTL, *qSF*_*n*_ *5* for SF_n_, overlapped with the flanking region previously reported (Li et al., 2018) (Supplementary Table S5), and the remaining five QTLs were newly identified in this study. The position range of one QTL, *qSF*_*ht*_ *7* for SF_ht_, overlapped with a previously reported flanking region (Ye et al., 2012) (Supplementary Table S5), and the remaining one, QTL *qSF*_*ht*_ *5*, was newly identified in this study. The position range of four QTLs for RSF overlapped with the flanking regions of three QTLs and one previously reported gene (*OsHTAs*) (Jagadish et al., 2010; Ye et al., 2012; Liu et al., 2016; Li et al., 2018) (Supplementary Table S5), and the remaining QTL, *qRSF2,* was newly identified in this study. In conclusion, these results indirectly demonstrate the reliability of our GWAS results.

The qRT-PCR results showed that only gene, *LOC_Os09g38500*, had a significant expression difference among 16 candidate genes (Figure 4F, Supplementary Figure S2). The expression of *LOC_Os09g38500* in the accessions with large RSF showed a tendency of increasing first (at a heat stress time from 08:30 a.m. to 11:30 a.m.) and then decreasing (at a heat stress time from 12:30 p.m. to 17:30 p.m.). These results again confirmed that heat tolerance in rice is a stress response adaptation for temperature change during the long period of domestication. Before the heat of day arrives, the heat-resistant rice have already adjusted accordingly.

In this study, gene *LOC_Os09g38500* was identified as a new candidate gene by GWAS and qRT-PCR. The full length of gene *LOC_Os09g38500* is 2,936 bp including three exons and three introns. Gene *LOC_Os09g38500* encodes a mitochondrial glycoprotein (Supplementary Table S4). We speculated that this protein could protect the mitochondrial membrane system from damage at high temperatures and thereby maintain the plant’s energy needs. According to the haplotype classification, the average RSF of Hap 1, Hap 2, and Hap 3 was 32.6, 40.5, and 43.9%, respectively. Hap 2 and Hap 3 can increase heat tolerance by 7.9 and 11.3%, respectively. Therefore, *LOC_Os09g38500* has potential application value in improving the molecular breeding of heat tolerance in rice.

## Data Availability

The data presented in the study are deposited in the Sequence Read Archive (SRA), NCBI with the accession code PRJNA554986.

## Abbreviations

ANOVA, analysis of variance; BIL, backcross inbred line; CSSL, chromosome segment substitution line; CV, coefficients of variation; DH, doubled haploid; GWAS, genome-wide association study; HTAS, heat tolerance at seedling stage; LD, linkage disequilibrium; MAF, minimum allele frequency; MLM, mixed linear model; NFS, the number of filled spikelets; PVE, phenotypic variation explained; qRT-PCR, quantitative real-time polymerase chain reaction; QTL, quantitative trait loci; RIL, recombinant inbred line; RSF, relative spikelet fertility; SNP, single nucleotide polymorphism; SF, spikelet fertility; SFn, spikelet fertility in natural condition; SFht, spikelet fertility in high temperature condition; TNS, the total number of spikelets; TOGR1, thermotolerant growth required 1; TT1, thermo-tolerance 1.
