# Supplementary material for: QTL mapping and identification of candidate genes using a genome-wide association study for heat tolerance at anthesis in rice (Oryza sativa L.)
Source: Front Genet. 2022 Sep 15;13:983525. doi: 10.3389/fgene.2022.983525 (PMC9520461; doi:10.3389/fgene.2022.983525)
Supplement: Supplementary file 1 [file Table4.DOC]

**Table S4.** Candidate gene annotation in the region 22.11–22.19 Mb associated with relative spikelet fertility.

| Number | MSU ID | Position | Annotation |
| --- | --- | --- | --- |
| 1 | LOC_Os09g38410 | 22,107,352-22,111,699 | Putative protein |
| 2 | LOC_Os09g38420 | 22,114,200-22,119,990 | cysteinyl-tRNA synthetase |
| 3 | LOC_Os09g38429 | 22,120,583-22,120,834 | hypothetical protein |
| 4 | LOC_Os09g38440 | 22,130,437-22,135,245 | Expressed protein |
| 5 | LOC_Os09g38450 | 22,134,460-22,137,041 | Expressed protein |
| 6 | LOC_Os09g38460 | 22,138,537-22,139,595 | Expressed protein |
| 7 | LOC_Os09g38480 | 22,145,704-22,147,511 | Expressed protein |
| 8 | LOC_Os09g38490 | 22,149,739-22,151,816 | Expressed protein |
| 9 | LOC_Os09g38500 | 22,152,674-22,155,609 | mitochondrial glycoprotein |
| 10 | LOC_Os09g38510 | 22,156,926-22,159,600 | Purine permease |
| 11 | LOC_Os09g38520 | 22,160,877-22,163,804 | DOMON domain containing protein |
| 12 | LOC_Os09g38530 | 22,168,446-22,172,434 | transmembrane 9 superfamily member |
| 13 | LOC_Os09g38540 | 22,172,840-22,174,402 | plastocyanin-like domain containing protein |
| 14 | LOC_Os09g38550 | 22,173,732-22,178,361 | protein phosphatase 2C |
| 15 | LOC_Os09g38560 | 22,181,587-22,183,290 | OsRCI2-11 - Hydrophobic protein OSR8 |
| 16 | LOC_Os09g38570 | 22,188,092-22,191,842 | transcription factor like protein, |
