# Supplementary figures and images for: QTL mapping and identification of candidate genes using a genome-wide association study for heat tolerance at anthesis in rice (Oryza sativa L.)
Source: Front Genet. 2022 Sep 15;13:983525. doi: 10.3389/fgene.2022.983525 (PMC9520461; doi:10.3389/fgene.2022.983525)

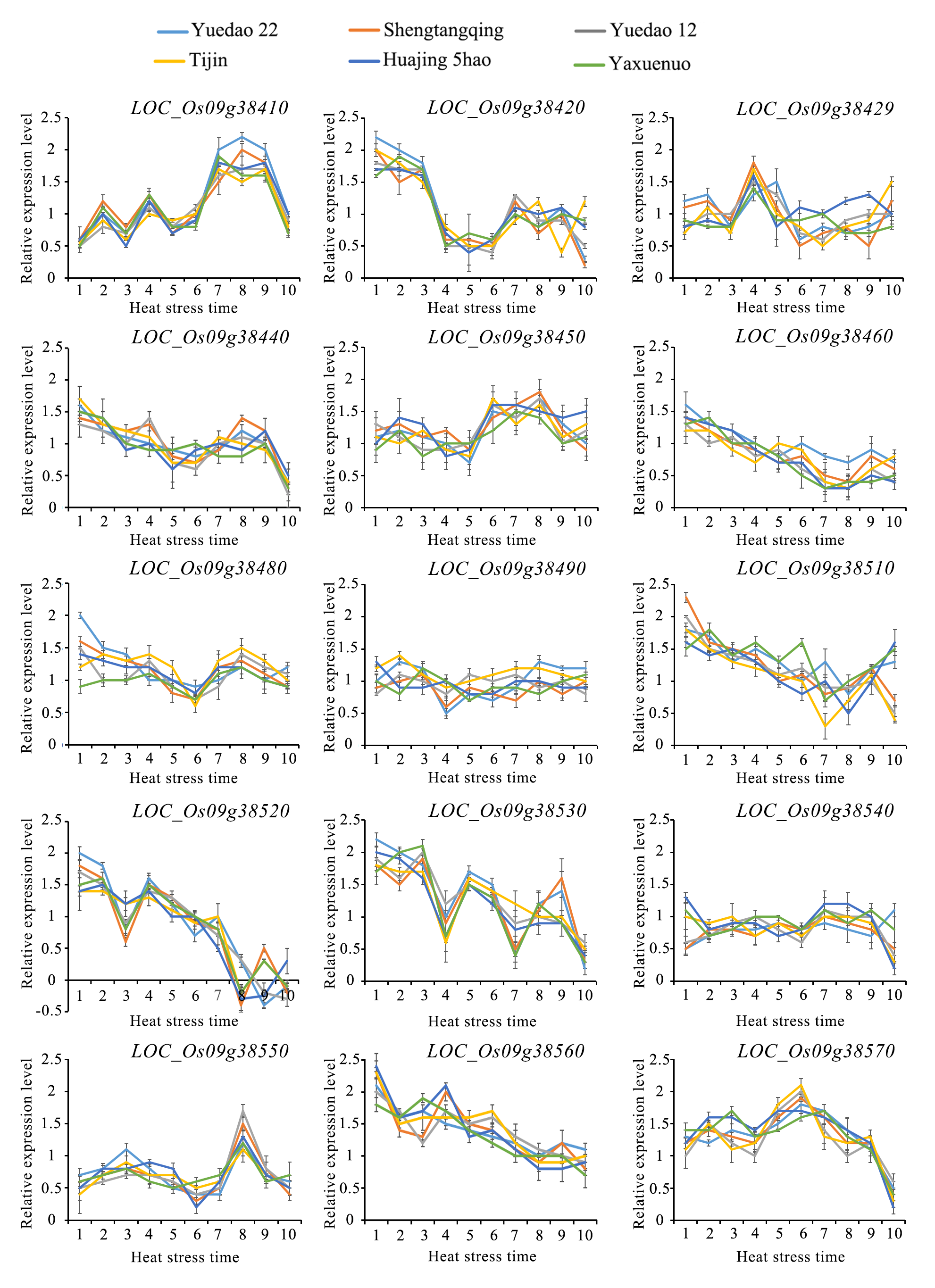

Supplement: Supplementary file 3 [file Image2.TIF]

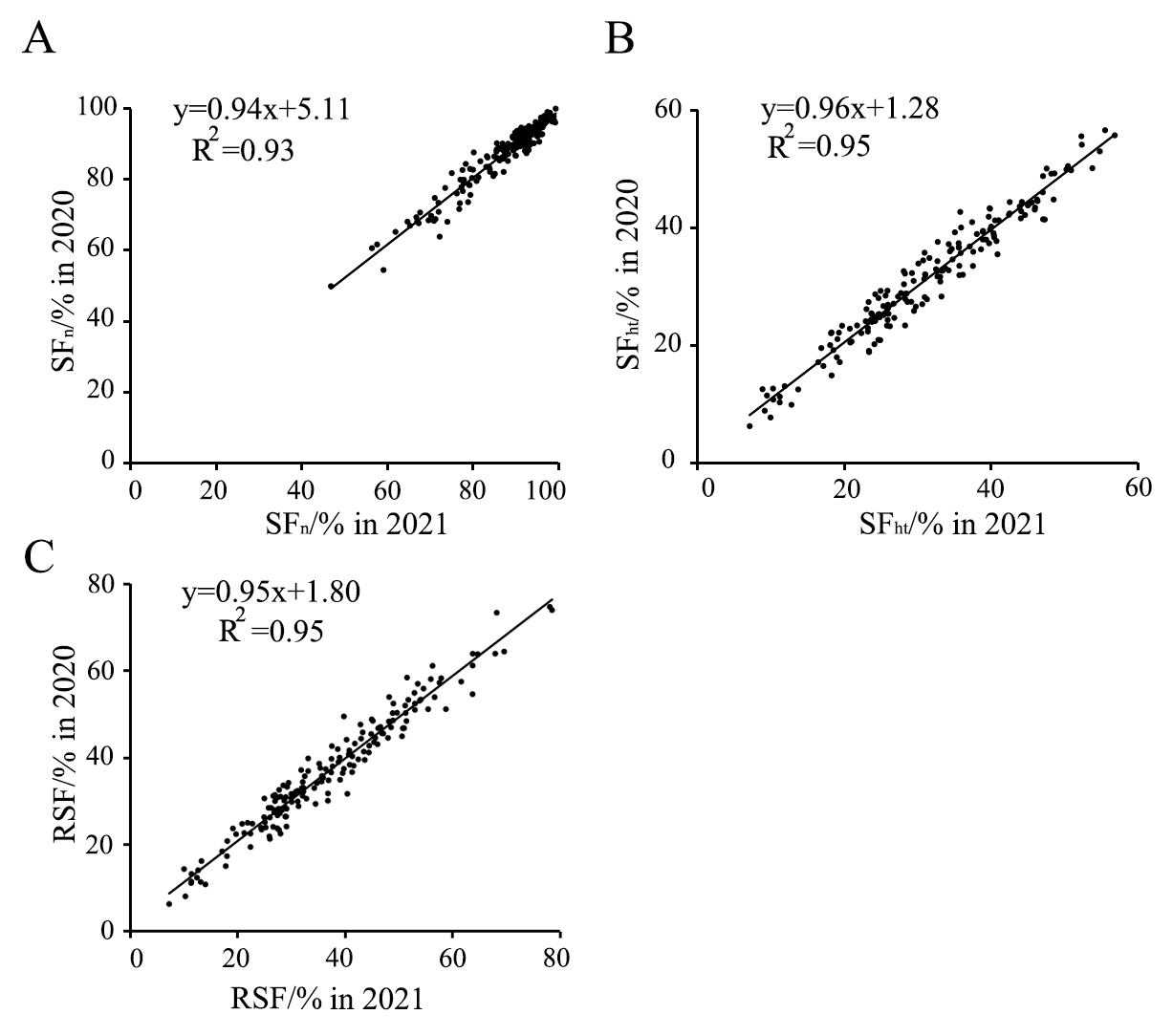

Supplement: Supplementary file 5 [file Image1.TIF]
