# Supplementary material for: QTL mapping and identification of candidate genes using a genome-wide association study for heat tolerance at anthesis in rice (Oryza sativa L.)
Source: Front Genet. 2022 Sep 15;13:983525. doi: 10.3389/fgene.2022.983525 (PMC9520461; doi:10.3389/fgene.2022.983525)
Supplement: Supplementary file 6 [file Table5.DOC]

**Table S5.** The results of QTLs detected in this study overlapped with the QTLs/Genes reported previously.

| QTL name | Chr. | Position range /bp | Known gene^a^ | Known QTL^a^/  association locus^a^ | Flanking region /bp^a^ | Reference |
| --- | --- | --- | --- | --- | --- | --- |
| *qSF_n_* | 5 | 7,797,279-7,997,279 |  | *qNS5* | 7,808,369-11,520,428 | Li et al. 2018 |
| *qSF_ht_* | 7 | 28,565,880-28,765,880 |  | *qHTSF7* | 28,467,201-28,910,103 | Ye et al. 2012 |
| *qRSF1* | 1 | 18,960,745-19,160,745 |  | *qRRS1* | 19,072,021-23,040,325 | Li et al. 2018 |
| *qRSF9.1* | 9.1 | 9,280,313-9,480,313 | *OsHTAs* |  | 9,444,998-9,449,781 | Liu et al. 2016 |
| *qRSF9.2* | 9.2 | 22,059,984-22,259,984 |  | *qHTSF9.1* | 22,016,036-22,445,124 | Ye et al. 2012 |
| *qRSF10* | 10 | 14,852,621-15,052,621 |  | *qRSF10.1* | 14,976,031-22,272,525 | Jagadish et al. 2010 |

^a^The gene name, QTL name and the physical position (bp) was inferred from the database of Gramene website (http://www.gramene.org/markers/), BLAST (http://blast.ncbi.nlm.nih.gov/Blast.cgi) and the China Rice Data Center database (http://www.ricedata.cn/gene/list/1499.htm).
