# Supplementary material for: QTL mapping and identification of candidate genes using a genome-wide association study for heat tolerance at anthesis in rice (Oryza sativa L.)
Source: Front Genet. 2022 Sep 15;13:983525. doi: 10.3389/fgene.2022.983525 (PMC9520461; doi:10.3389/fgene.2022.983525)
Supplement: Supplementary file 7 [file Table2.DOC]

**Table S2.** The sequences of primers used for qRT-PCR.

| Gene name | Forward primer sequence (5’-3’) | Reverse primer sequence (5’-3’) |
| --- | --- | --- |
| UBQ | ACCCTGGCTGACTACAACATC | AGTTGACAGCCCTAGGGTG |
| LOC_Os09g38410 | TGGAGACACAGAGGACGAGA | ACATATGCACGCTTGTTCAC |
| LOC_Os09g38420 | TGCAGCCTGGAGAAACATAA | TATGCAATTGGCGGGGATGT |
| LOC_Os09g38429 | GCCAGGAATGGAAAGCTACGA | GATCTACCTATGTCGCCTCCT |
| LOC_Os09g38440 | TGCGGTTCAAGCCTGTGTAA | CCACAGACCACATGAACCCA |
| LOC_Os09g38450 | TCCTTGTTCTCGCTGCCATT | GGAGCTCTTGTACGCCAAGT |
| LOC_Os09g38460 | TGGGTTACCTGGACAACGTG | CAACGAAGGCCACTACCTGT |
| LOC_Os09g38480 | GCCTTTGTTGATCTCTGGCG | TCTGACAACGGTGAGACGAC |
| LOC_Os09g38490 | CCGCAGGTCTCCTACATCTG | AATCCCTCTGTAAGCTGGCG |
| LOC_Os09g38500 | TTGCACGACTACATGGCGAA | CAAGGGATTGGATTCCCTGAT |
| LOC_Os09g38510 | ACAGGATCTTGAAGCCGCTC | GAGACCTCATCAGAGGCAGC |
| LOC_Os09g38520 | GGCTCCGAGTACTACATGGC | AAACCTTGTCAGGGCACACA |
| LOC_Os09g38530 | TCCCTTCCTTTCTGCAAGCC | AGTGCACAACTTACTCCGGT |
| LOC_Os09g38540 | TTCATCCCGCTCAACGACTC | CCATCCTGTCGCTGCTTTCT |
| LOC_Os09g38550 | TGACTGACGTACTCGACCCT | GTCTGCACCGCTTTTTACCC |
| LOC_Os09g38560 | ACTCTGCTACATCTGCCTGTG | CACGAGGGGCGGAAAAGATA |
| LOC_Os09g38570 | CTCTGCTACATCTGCCTGTGT | AGCCAAGGATGGTGAGAAGC |
